# Conditionally Reprogrammed Cells from Patient-Derived Xenograft to Model Neuroendocrine Prostate Cancer Development

**DOI:** 10.3390/cells9061398

**Published:** 2020-06-04

**Authors:** Xinpei Ci, Jun Hao, Xin Dong, Hui Xue, Rebecca Wu, Stephen Yiu Chuen Choi, Anne M. Haegert, Colin C. Collins, Xuefeng Liu, Dong Lin, Yuzhuo Wang

**Affiliations:** 1 Vancouver Prostate Centre, Department of Urologic Sciences, Faculty of Medicine, University of British Columbia, Vancouver, BC V6H 3Z6, Canada; xci@prostatecentre.com (X.C.); jhao@prostatecentre.com (J.H.); ahaegert@prostatecentre.com (A.M.H.); ccollins@prostatecentre.com (C.C.C.); 2 Department of Experimental Therapeutics, BC Cancer Agency, Vancouver, BC V5Z 1L3, Canada; xdong@bccrc.ca (X.D.); hxue@bccrc.ca (H.X.); rwu@bccrc.ca (R.W.); schoi@bccrc.ca (S.Y.C.C.); 3 Department of Pathology, Center for Cell Reprogramming, Georgetown University Medical Center, Washington, DC 20057, USA; 4 Department of Oncology, Lombardi Comprehensive Cancer Center, Georgetown University Medical Center, Washington, DC 20057, USA

**Keywords:** neuroendocrine prostate cancer, patient-derived xenograft, conditional reprogramming culture

## Abstract

Neuroendocrine prostate cancer (NEPC) is a lethal subtype of prostate cancer. It develops mainly via NE transdifferentiation of prostate adenocarcinoma in response to androgen receptor (AR)-inhibition therapy. The study of NEPC development has been hampered by a lack of clinically relevant models. We previously established a unique and first-in-field patient-derived xenograft (PDX) model of adenocarcinoma (LTL331)-to-NEPC (LTL331R) transdifferentiation. In this study, we applied conditional reprogramming (CR) culture to establish a LTL331 PDX-derived cancer cell line named LTL331_CR_Cell. These cells retain the same genomic mutations as the LTL331 parental tumor. They can be continuously propagated in vitro and can be genetically manipulated. Androgen deprivation treatment on LTL331_CR_Cells had no effect on cell proliferation. Transcriptomic analyses comparing the LTL331_CR_Cell to its parental tumor revealed a profound downregulation of the androgen response pathway and an upregulation of stem and basal cell marker genes. The transcriptome of LTL331_CR_Cells partially resembles that of post-castrated LTL331 xenografts in mice. Notably, when grafted under the renal capsules of male NOD/SCID mice, LTL331_CR_Cells spontaneously gave rise to NEPC tumors. This is evidenced by the histological expression of the NE marker CD56 and the loss of adenocarcinoma markers such as PSA. Transcriptomic analyses of the newly developed NEPC tumors further demonstrate marked enrichment of NEPC signature genes and loss of AR signaling genes. This study provides a novel research tool derived from a unique PDX model. It allows for the investigation of mechanisms underlying NEPC development by enabling gene manipulations ex vivo and subsequent functional evaluations in vivo.

## 1. Introduction

Prostate cancer (PCa) is the second most commonly diagnosed cancer in men worldwide, with 1.3 million new cases and 360,000 deaths reported in 2018 [1]. Androgen deprivation therapy (ADT) and more potent androgen receptor pathway inhibitors (ARPI) have brought significant improvements to the management of advanced PCa over the past decades. However, treatment resistance with the acquisition of cellular plasticity inevitably occurs [2,3]. Unfortunately, the study of the relevant underlying mechanisms is limited by a lack of models. Developing better models and extending the applications of existing ones are thus fundamental in aiding novel therapeutic development.

Neuroendocrine prostate cancer (NEPC) is an extremely aggressive subtype of PCa. While de novo cases are extremely rare (0.05% of PCa) [4], its incidence reaches 17–25% of patients receiving ARPI treatment [5,6,7]. With no effective therapy, NEPC has the worst prognosis of all PCa subtypes. The median survival time from diagnosis is less than 1 year [5,8,9]. One of the major hurdles in studying NEPC is the lack of clinically relevant models. Our laboratory has established a unique, first-in-field adenocarcinoma-to-NEPC transdifferentiation patient-derived xenograft (PDX) model known as LTL331/331R [10]. Upon host castration, the primary adenocarcinoma (LTL331) initially regresses but relapses within a few months as typical NEPC (LTL331R). This fully recapitulates disease progression in the donor patient [10,11]. Utilizing this model, we have performed genomic, transcriptomic, and epigenetic analyses over the course of the whole transdifferentiation process and have identified critical upregulated molecules such as HP1α, PEG10, DEK, CBX2, ONECUT2, SRRM4, BRN2, and long-noncoding RNA MIAT during NEPC development [11,12,13,14,15,16,17,18]. However, using PDX models for molecular functional studies and high-throughput genetic and drug screening is still hindered by a lack of ex vivo primary cells as an interface. Organoid cultures have been a partial solution to this challenge but suffer from low success rates in practice [19]. As such, an easy-to-use method with a high success rate for culturing primary cells is critically needed to take advantage of existing PDX models.

Conditional reprogramming (CR) is a method of co-culturing primary cells derived from normal and tumor epithelial tissues with irradiated mouse 3T3-J2 feeder cells using culture media supplemented with the ROCK inhibitor Y27632 [20,21]. This special culture condition confers indefinite propagation ability and adult stem-like (but not pluripotent) characteristics on primary cells without any additional genetic manipulation [20,22]. The tumor-derived CR cells retain the heterogeneity and molecular characteristics of the parental tumor, thereby providing a high-fidelity platform for basic research and drug screening [23,24,25,26,27,28]. The stem-like and dedifferentiated status of primary cells upon CR culture is also reversible. Once CR components are withdrawn from the culture or when cells are grafted into mice in vivo, the primary cells will revert back to their committed cell fate [20,22]. Here, we applied the CR culturing technique to our LTL331 model to establish primary cells. We then evaluated their biological features both in vitro and in vivo.

## 2. Materials and Methods

### 2.1. Establishment and Culture of Primary CR Cells

Primary CR cell lines were established following protocols reported in previous studies [20,21]. Briefly, PDX tumor tissues were collected from the host mouse renal capsule site while avoiding mouse renal tissue contamination. Fresh tissues were then minced in Hank’s Balanced Salt Solution (Thermo Fisher, Waltham, Massachusetts) on ice and digested with 5 mg/mL collagenase I (Thermo Fisher, Waltham, MA, USA) dissolved in 10 mL DMEM/F12 (Thermo Fisher, Waltham, MA, USA) medium supplemented with 10 μM Y27632 (Stemcell Technologies, Vancouver, BC, Canada). The digested tissue was passed through a cell strainer (100 μm, BD Biosciences, San Jose, CA, USA) to remove large tissue pieces. The remaining cells were further digested with 4 mL TrypLE (Thermo Fisher, Waltham, MA, USA) supplemented with 10 µM Y27632 and filtered through another cell strainer (40 μm, BD Biosciences, San Jose, CA, USA). The cells were then counted and plated on 100 mm cell culture dishes (Thermo Fisher, Waltham, MA, USA) with irradiated Swiss 3T3-J2 (ATCC, Manassas, VA, USA) feeder cells (90% confluence, 30 Gys X-ray irradiation). To passage CR cells, old feeder cells were digested with 1 mL 0.05% Trypsin/EDTA reagent (Thermo Fisher, Waltham, MA, USA) and washed out with 10 mL PBS; CR cells were then digested with 4 mL TrypLE. The digestion was neutralized with 5 mL complete F medium and the cells were resuspended in 5 mL F medium. Primary CR cells were maintained in 10 mL F medium containing the following reagents: DMEM/F12 (3:1 (v/v), Thermo Fisher, Waltham, MA, USA), 5% fetal bovine serum (Thermo Fisher, Waltham, MA, USA), 0.4 μg/mL hydrocortisone (Sigma-Aldrich, St. Louis, MO, USA), 5 μg/mL insulin (Sigma-Aldrich, St. Louis, MO, USA), 8.4 ng/mL cholera toxin (Sigma-Aldrich, St. Louis, MO, USA), 10 ng/mL epidermal growth factor (Sigma-Aldrich, St. Louis, MO, USA), 24 μg/mL adenine (Sigma-Aldrich, St. Louis, MO, USA), and 10 μM Y-27632. 3T3-J2 feeder cells were cultured in 100 mm cell culture dishes (Thermo Fisher, Waltham, MA, USA) with 10 mL DMEM medium containing 10% bovine calf serum (ATCC, Manassas, Virginia, VA, USA).

### 2.2. PDX Models and Renal Capsule Xenografting

As previously described, PDX tumor lines were grafted in nonobese diabetic/severely combined immunodeficient (NOD/SCID) mice. Host castration was achieved by surgical removal of the testes [10]. This study followed the ethical guidelines stated in the Declaration of Helsinki. Specimens were obtained from patients with their informed written consent following the protocol (#H09-01628) approved by the Institutional Review Board of the University of British Columbia (UBC). Animal studies were completed under protocol # A17-0165 as approved by UBC Animal Care and Use Committee. For primary cell xenografting, 1 × 10^7^ cells were mixed with rat tail collagen (5 mg/mL, house made) at a 1:1 ratio for a final volume of 100 μl before grafting under the mouse renal capsule. Tumor growth and mouse status were actively monitored, and tumor images were captured using the Vevo 3100 imaging system (Visualsonics, Toronto, ON, Canada). Xenograft tumors, together with other host organs, were collected upon reaching the humane endpoint based on health status evaluations or when the tumor size reaches 1000 mm^3^.

### 2.3. RNA Sequencing and Bioinformatic Analyses

Tissues were homogenized on the Precellys (Bertin Instruments, Montigny-le-Bretonneux, France) in Maxwell kit homogenization buffer (Promega, Madison, WI, USA). Total RNA was extracted using the Maxwell RSC Simply RNA Tissue kit (Promega, Madison, WI, USA). RNA quality was assessed by Agilent Tapestation (Agilent, Santa Clara, CA, USA) and Qubit quantitation (Thermo Fisher, Waltham, MA, USA). RNA libraries were prepared from 200 ng of total RNA using the Illumina TruSeq Stranded mRNA Library Prep Kit (Illumina, San Diego, CA, USA. Library quality was assessed by Agilent Tapestation, Qubit, and qPCR quantitation. Libraries were pooled and sequenced on the NextSeq 500 System (Illumina, San Diego, CA, USA) with the aim of at least 20M reads per library. Raw data from RNA sequencing was processed through BBtools to extract human reads from potential host mouse contamination [29]. Sequence alignment and variant calling were performed against the reference human genome (UCSC hg19). Genomic indexes were generated and paired-end sequence reads were aligned using STAR aligner [30]. Gene expression data were normalized with DESeq2 1.16.1 [31]. Picard (“Picard Toolkit.” 2019. Broad Institute, GitHub Repository. http://broadinstitute.github.io/picard/; Broad Institute) was then used to add read groups, sort, mark duplicates, and create index files. Mutations and indels were called using the Genome Analysis Toolkit (GATK) [32]. ANNOVAR software (https://doc-openbio.readthedocs.io/projects/annovar/en/latest/user-guide/download/) was used to annotate variants [33]. Groups of mutations were visualized in R using the maftools package [34]. Python scripts used for calling transcripts mutations were provided in supplementary information (SI.1). All RNA-seq profiles have been deposited to gene expression omnibus with accession number GSE149091.

Gene set enrichment analysis (GSEA) was used in this study to determine whether a defined set of genes shows significant concordant differences between two groups [35]. Normalized gene expression values of the whole transcriptome were used for analysis. A pre-ranked gene list was generated by ranking the gene expression difference between LTL331_CR_Cell and the parental LTL331 tumor from high to low. Unbiased analysis was performed using the latest MSigDB database for each collection (https://www.gsea-msigdb.org/gsea/msigdb/index.jsp). The 331 CAS 12W_UP 100 and 331 CAS 12W_DOWN 100 gene sets were manually generated using the top 100 upregulated and downregulated genes identified when comparing LTL331 at 12-weeks post-castration to the parental LTL331 tumor (Table S1) [11]. Gene set permutation was employed. False discovery rate (FDR) q-values were calculated using 1000 permutations, and a gene set was considered significantly enriched if its normalized enrichment score (NES) has an FDR q below 0.25.

The NE and AR scores were calculated as previously reported [7,12]. Briefly, a score was assigned to each sample by multiplying the gene weight with the normalized RNA expression value. The values were then added for each gene. The absolute values of the sum value were log-transformed and multiplied by the sign of the sum value.

### 2.4. Lentivirus Production and Generation of Stable Cell Lines

Lentiviruses expressing mCherry were produced in 293T cells following established protocols [36]. Culture medium containing lentiviruses were collected 72 h after virus packaging. After filtration, virus-contained medium were added to the culture medium of primary CR cells. Puromycin (Thermo Fisher, Waltham, MA, USA) was used at 1 μg/mL to select for infected cells and to maintain stable cell lines.

### 2.5. Immunohistochemistry Staining

Formalin-fixed paraffin-embedded (FFPE) tissue sections were stained manually using our established protocols. Briefly, tissue sections on glass slides were deparaffinized in xylene, rehydrated in graded ethanol solutions, and washed in tap water. Antigen retrieval was performed by boiling the slides in a citrate buffer (10 mM trisodium citrate, pH 6.0). After a 10-min treatment with 3% H_2_O_2_, tissue sections were blocked with 5% normal goat serum in Tris-buffered saline with 0.1% Tween-20 for 1 h at room temperature. They were then incubated with primary antibodies at 4 °C overnight. The next day, biotinylated secondary antibodies (Vector Laboratories, Foster City, CA, USA) were added for 30 min at room temperature. The slides were then incubated with avidin–biotin complex (Vector Laboratories, Foster City, CA, USA) for another 30 minutes at room temperature. Finally, after the application of DAB chromogen, tissue sections were stained with hematoxylin, dehydrated, and mounted [10]. The primary antibodies used in this study are: pan-CK (DaKo, Santa Clara, CA, USA), Ki67 (Clone MIB-1, DaKo, Santa Clara, CA, USA), CD56 (Clone MRQ-42, Sigma-Aldrich, St. Louis, MO, USA), PSA (ab76113, Abcam, Cambridge, MA, USA), and AR (ab108341, Abcam, Cambridge, MA, USA). The histopathology and staining intensities were evaluated by two independent pathologists.

### 2.6. Quantitative Real Time-PCR (qRT-PCR)

For cultured cells, total RNA was extracted using TRIzol (Life technology, Waltham, MA, USA). For tumor tissues, total RNA was extracted using the miRNeasy kit (Qiagen, Valencia, CA, USA). First-strand cDNA was synthesized from 1 μg of total RNA using the QuantitectTM reverse transcription kit (Qiagen, Valencia, CA, USA). RT-PCR was performed using SYBR Green reagent and the Applied Biosystems ViiA-7 Real Time PCR system (Applied Biosystems, Foster City, CA, USA). The qRT-PCR primers used in this study are as follows: AR_forward: 5′- TCTTGTCGTCTTCGGAAATGT; AR_reverse: 5′- AAGCCTCTCCTTCCTCCTGTA; KLK3_forward: 5′- CACCTGCTCGGGTGATTCTG; KLK3_reverse: 5′- CCACTTCCGGTAATGCACCA. GAPDH forward: 5′- CACCAGGGCTGCTTTTAACTC; GAPDH reverse: 5′- GACAAGCTTCCCGTTCTCAG. Relative gene expression was calculated using the 2-∆∆ct method with GAPDH as an internal reference gene.

### 2.7. Statistical Analysis

The Student t-test was used to determine statistical significance between groups with discrete measurements. Any differences with *p*-values lower than 0.05 is regarded as statistically significant, indicated by * for *p* < 0.05, ** for *p* < 0.01, and *** for *p* < 0.001. In GSEA, the nominal *p*-value estimates the statistical significance of the enrichment score for a single gene set. The FDR q-value is the estimated probability that a gene set with a given NES represents a false-positive finding. A nominal *p*-value less than 0.05 and a FDR q-value less than 0.25 is considered statistically significant [35].

## 3. Results

### 3.1. Establishment and Application of Conditionally Reprogrammed Cells from PDX

**Figure 1:**
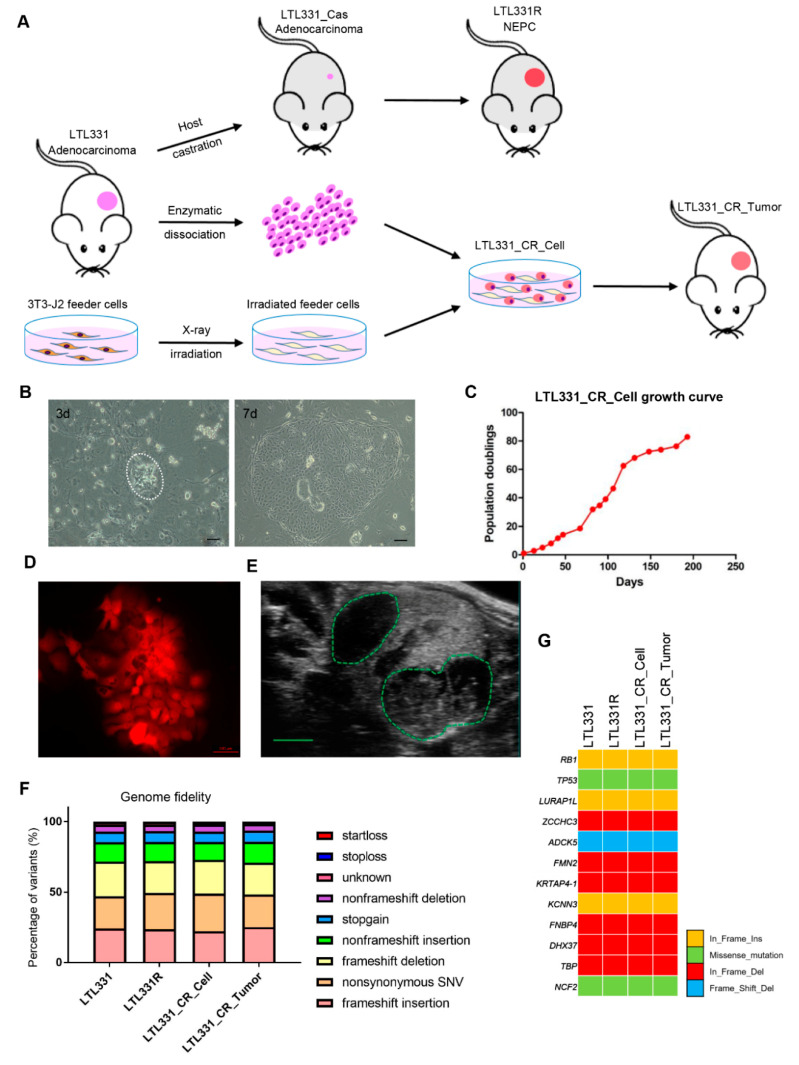
Establishment, application, and molecular characterization of conditionally reprogrammed cells derived from the LTL331 PDX. (**A**) Schematic showing the conditional reprogramming (CR) culture establishment process. The LTL331 prostatic adenocarcinoma PDX can transdifferentiate to neuroendocrine prostate cancer (NEPC) (LTL331R) after host mouse castration-induced tumor regression (LTL331_Cas) [10]. Fresh tumor tissue from LTL331 was enzymatically dissociated into single cells. The cells were then cocultured with irradiated 3T3-J2 feeder cells to establish LTL331_CR_Cells. The CR cells were grafted under the renal capsule of host mice to establish CR cells-derived xenografts, namely LTL331_CR_ Tumor. (**B**) Representative images of LTL331_CR_Cells. Under the light microscope, small colonies of epithelial tumor cells can be observed 3 days after initial plating as highlighted by the white dash (left panel). Faster growing large colonies can be observed after one week as shown in the right panel. Scale bar, 200 μm. (**C**) Growth curve of LTL331_CR_Cell. The CR cells were serially passaged and the cell numbers were recorded at each passage until the culture was terminated. A growth curve of population doublings versus time (days) is plotted. (**D**) Representative image of LTL331_CR_Cell following genetic manipulation. Cells were transduced with lentivirus to express the mCherry fluorescent protein. Scale bar, 100 μm. (**E**) Representative image of LTL331_CR_Tumor growing under the renal capsule of a mouse. The green dash highlights two tumors grafted in one kidney. Scale bar, 2 mm. (**F**–**G**) Mutation analysis of expressed transcripts across the LTL331-derived models. Mutation type and frequency was analyzed with RNA-seq data. The percentage of each mutation type is stacked into one bar, with different types indicated by the corresponding colors (**F**). Representative gene mutations are shown (**G**).

We applied the CR culture method to establish LTL331 primary cells with the hope of providing an ex vivo interface to study NEPC development. In brief, freshly collected LTL331 tumor was enzymatically disassociated. The cells were then plated onto irradiated 3T3-J2 feeder cells and cultured using CR culture F medium (Figure 1A). The established primary cell line is designated LTL331_CR_Cell hereafter. LTL331_CR_Cells were re-grafted under the renal capsules of male mice to determine in vivo tumor growth and their corresponding histopathological characteristics (i.e., overall tumor structure, microscopic appearance of cancer cells, and cell-type marker expressions) (Figure 1A). In vitro, LTL331_CR_Cells formed colonies with epithelial morphology 3 days after initial plating and rapidly reached proliferative confluence in one week (Figure 1B). The average population doubling time was 72 h as estimated from 20 passages over 200 days (Figure 1C). This doubling time is three-fold shorter than that of the parental tumor (9 days) [10]. These CR cells were amenable to genetic manipulation as demonstrated by fluorescent protein labeling using lentiviral transduction (Figure 1D). We also re-grafted LTL331_CR_Cells (passage 10) under the renal capsules of male NOD/SCID mice (Figure 1E). This grafting site is where the parental LTL331 PDX tumor can survive and grow well. Tumors arising from LTL331_CR_Cells are designated LTL331_CR_Tumor hereafter. To confirm that LTL331_CR_Cell and LTL331_CR_Tumor conserve the genomic features of the parental LTL331 PDX, we analyzed the gene transcript mutation profiles using RNA-seq data. Both the whole transcript alteration landscape and the targeted gene mutation status show that LTL331_CR_Cell and LTL331_CR_Tumor share identical genomic alterations with parental LTL331 PDX (Figure 1F,G and Figure S1).

### 3.2. LTL331_CR_Cell Is Androgen Independent with Stem-like Features

**Figure 2:**
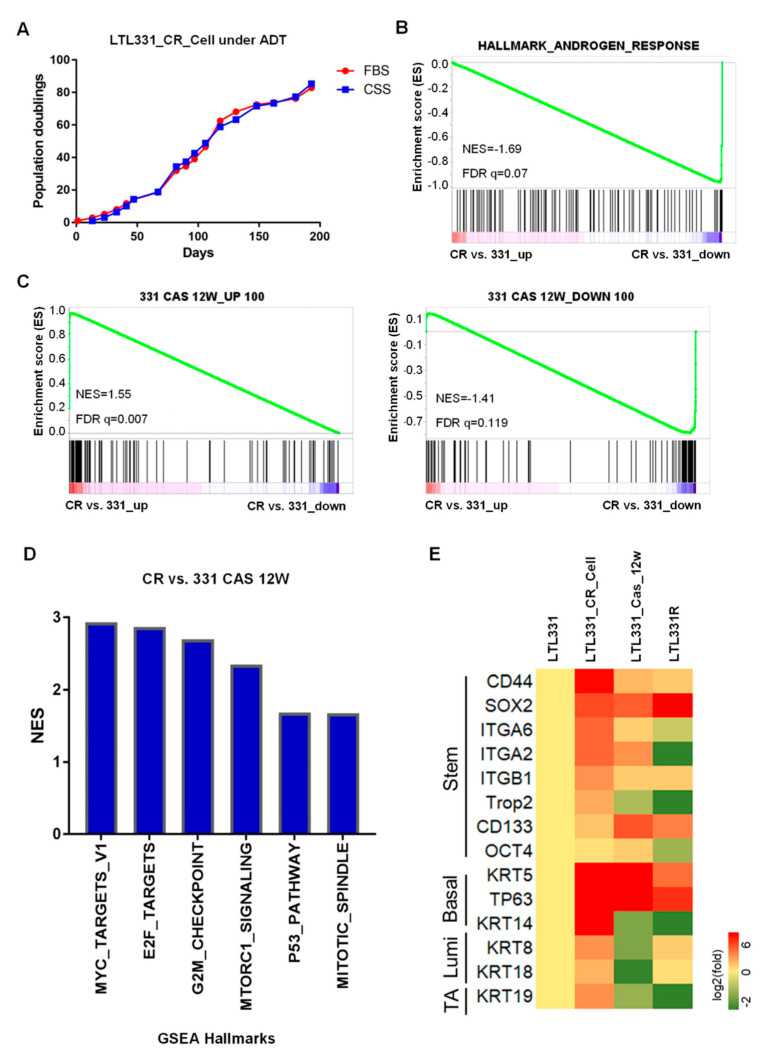
LTL331_CR_Cell is androgen independent with stem-like features. (**A**). Growth curve of LTL331_CR_Cells under androgen deprivation therapy (ADT) conditions. CR cells were cultured in medium containing charcoal stripped serum (CSS) to mimic ADT. The cells were passaged and cell numbers were recorded at each passage. The blue curve shows cells grown in CSS-containing medium, while the red curve shows cells grown in normal medium containing FBS. The red curve is the same as Figure 1C. (**B**) Inactivation of AR signaling in LTL331_CR_Cells. Transcriptomic analysis using gene set enrichment analysis (GSEA) shows that androgen response is downregulated in LTL331_CR_Cells compared to the parental LTL331. (**C**) The transcriptome of LTL331_CR_Cells partially resembles that of LTL331 post-castration. The top 100 upregulated and downregulated genes from 12-week post-castrated LTL331 were utilized as gene sets (i.e., 331 CAS 12W_UP 100, 331 CAS 12W_DOWN 100). GSEA comparing LTL331_CR_Cells to the parental LTL331 shows that a very similar set of castration-response genes are upregulated (left panel) and downregulated (right panel). (**D**) GSEA shows that select cancer hallmarks are enriched in LTL331_CR_Cells compared to post-castrated LTL331. The y-axis represents normalized enrichment scores (NES). The nominal *p*-values of all gene sets are less than 0.05. (**E**) Heatmap showing stem cell and cell lineage marker expression in LTL331_CR_Cells. Log-2 expression fold changes of select stem, basal, luminal, and intermediate transient amplifying (TA) cell markers were compared between LTL331_CR_Cell, 12-week post-castrated LTL331, LTL331R, and the parental LTL331.

Since the parental LTL331 PDX tumor can undergo neuroendocrine trandifferentiation upon host castration, we applied androgen deprivation treatment to LTL331_CR_Cells using culture medium containing charcoal-stripped serum (CSS). Interestingly, we found that LTL331_CR_Cells in CSS-medium behaved similarly to those in complete medium with a nearly identical population doubling curve (Figure 2A). We then analyzed the transcriptomic profiles of LTL331_CR_Cells and the parental LTL331 PDX tumor. Gene set enrichment analysis (GSEA) showed that hallmark genes involved in androgen response were predominantly downregulated in LTL331_CR_Cells compared to parental PDX (Figure 2B), suggesting that AR signaling is inhibited in CR cells. We also validated the expression of *AR* and its critical target gene *KLK3* using qRT-PCR (Figure S2). Because the LTL331 tumor shows inhibited AR signaling post-castration [10], we compared the gene expression profile of LTL331_CR_Cells to that from LTL331 tumors 12 weeks after castration. GSEA showed that most of the top 100 upregulated and downregulated genes in post-castrated LTL331 compared to the parental tumor were also similarly increased or decreased in CR cells (Figure 2C). This suggests that the CR culture condition in vitro partially mimics castration in vivo. Host castration results in a dramatic reduction in LTL331 tumor volume [10]. Considering that LTL331_CR_Cells are highly proliferative in an androgen-independent manner, we further explored the potential pathways involved in facilitating CR cell growth. GSEA of cancer hallmarks show that MYC, E2F, P53, MTORC1 and cell cycle progression pathways are highly activated in CR cells (Figure 2D, Table S2). Previous studies have reported that CR culture can confer stem-like characteristics on primary cells [20,22]. We thus further analyzed stem cell and lineage marker [37,38,39] expression in LTL331_CR_Cells and a series of LTL331 PDX tumors. While only some stem cell markers (e.g., SOX2, CD133) and basal cell markers (e.g., KRT5, TP63) are upregulated in post-castrated LTL331 and relapsed NEPC tumor (LTL331R), all of the stem, basal, luminal, and intermediate transient amplifying cell markers were consistently upregulated in LTL331_CR_Cells. These data suggest that LTL331_CR_Cells have stem-like features.

### 3.3. LTL331_CR_Cells Give Rise to NEPC Tumors In Vivo

**Figure 3:**
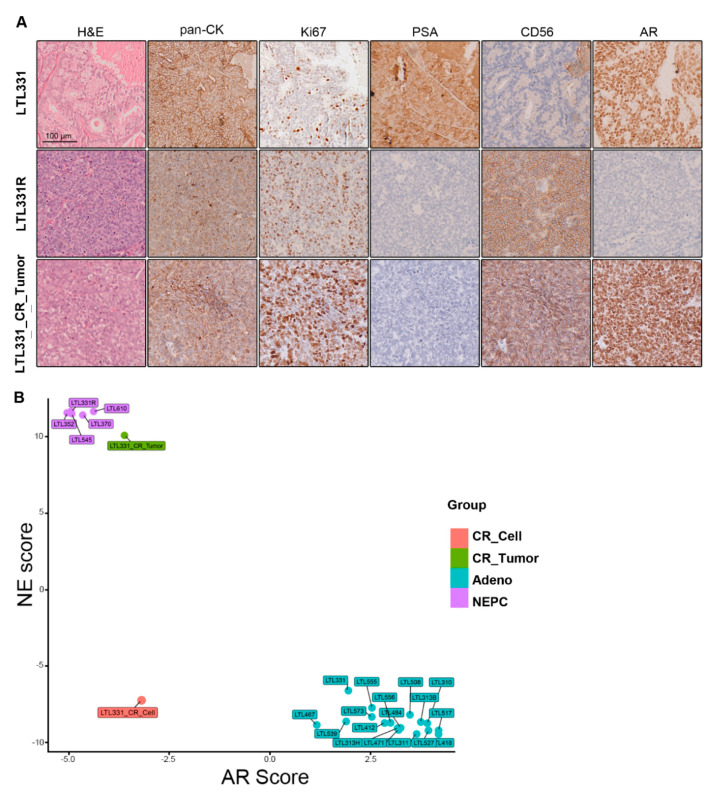
Characterization of LTL331_CR_Tumor as NEPC. (**A**) Representative images showing IHC staining of LTL331, LTL331R, and LTL331_CR_Tumor. H&E, epithelial tumor marker pan-CK, proliferation marker Ki67, NE marker CD56, AR, and AR signaling marker PSA were stained as indicated in the images. Scale bar, 100 μm. (**B**) AR and NE scores of LTL331_CR_Tumor. NE and AR scores were calculated using transcriptomic data from LTL331_CR_Tumor. The scores were also calculated for other independent PDX models and LTL331_CR_Cell. Sample types are differentially colored based on their histopathological classifications.

Previous studies have reported that CR cells, when implanted back into immunodeficient mice in vivo, can form tumors representing the original histopathology of the parental tumor and not the in vitro dedifferentiated state [20,21,27,40]. We thus grafted LTL331_CR_Cells under the renal capsules of male mice supplemented with testosterone in order to represent the human physiological environment [41,42]. Although we anticipated the formation of adenocarcinoma tumors similar to LTL331, the resultant LTL331_CR_Tumors were in fact NEPC. Histopathological characterization of the LTL331_CR_Tumor showed that tumor cells ubiquitously express the epithelial tumor marker pan-cytokeratin (pan-CK) and is mostly Ki67-positive (~80%) (Figure 3A). Compared to the parental LTL331 PDX (which is an adenocarcinoma tumor), LTL331_CR_Tumors do not have glandular structures, nor do they express intratumoral PSA as consistent with serum level measurements (Figure 3A). Notably, LTL331_CR_Tumor cells ubiquitously and strongly express the NE marker CD56 (Figure 3A). Thus, LTL331_CR_Tumors have gained NE histopathological features (NE positive, PSA negative [43]) and are more similar to the LTL331R NEPC tumor than the parental LTL331 adenocarcinoma tumor (Figure 3A). To further confirm the molecular features of the LTL331_CR_Tumor, we analyzed its transcriptomic profile. Compared to the parental LTL331 tumor, neuronal- and proliferation-associated signaling pathways are enriched in the LTL331_CR_Tumor (Figure S3A). Conversely, the AR pathway is inhibited as demonstrated by GSEA (Figure S3B) and the reduced expression of a panel of AR-target genes (Figure S3C). Consistent with its histopathological features, LTL331_CR_Tumor has a dramatically elevated NE signature score and a negative AR signaling score. These molecular features are also evident in five other independent NEPC PDX samples (Figure 3B). In agreement with NEPC histopathological and molecular characteristics, LTL331_CR_Tumor is also refractory to host mouse castration.

## 4. Discussion

In this study, we successfully established CR cells from our LTL331 PCa PDX model. As a proof-of-principle, this method can establish ex vivo models from existing PDX collections, thus expanding their applications. To the best of our knowledge, this is the first report of CR cultures being successfully used for PCa PDX models. Cell-based models are simple and cost- and time-efficient research tools [44]. However, there are only a few PCa cell lines in common use (e.g., LNCaP, PC-3, DU145). These are derived from metastatic sites of advanced PCa and do not reflect disease progression or heterogeneity [44,45]. Our laboratory (www.livingtumorlab.com) has established over 50 PCa PDX models covering hormone-naïve, castration-resistant, and neuroendocrine PCa subtypes [10]. These PDXs represent patient tumor characteristics and disease progression with high fidelity, thus enabling functional gene identifications and drug efficacy studies. However, to utilize PDXs for functional evaluations and molecular mechanism studies remains challenging because of the lack of matched ex vivo material for efficient genetic manipulations [44,46,47]. Organoid cultures represent a breakthrough in addressing this problem, but the success rate of stable organoid establishment remains low [19]. CR technology has been used for generating primary cells from various normal and cancer tissues, including patient-derived samples and PDX models [26,27,28,48]. This technique has the remarkable advantages in being easy-to-do, cost- and time-efficient, having high success rates, and being amenable to genetic manipulations. Consistent with other studies, we were able to rapidly establish proliferative primary cells within one week and were able to expand them indefinitely in principle. The average doubling time of the LTL331_CR_Cells were ~72 h, which is similar to that of the commonly used LNCaP PCa cell line (~60 h) [49]. However, this is much shorter than the doubling time of organoids (~ 7 days) and PDXs (~ 14 day) [10,19,50]. We were also able to perform genetic manipulations using standard protocols, which cannot be easily achieved in either organoids or PDXs [44,51]. We expect that more PCa PDX-derived CR cells can be established using this method, which will provide a powerful platform for studying PCa progression.

LTL331_CR_Cells show unexpected cellular plasticity, developing into NEPC in vivo. One feature of the CR technology is that it temporarily maintains cells in a stem-like state in vitro. Once regrafted in vivo, the CR cells re-differentiate into parental tissue morphology [22,27,40]. Previous studies generating CR cells from prostatic adenocarcinoma tissues have confirmed this phenomenon [21,28], as exemplified in Figure S4. While LTL331_CR_Cells also express stem-like markers and exhibit an intermediate transient-amplifying status (positive for both basal and luminal markers), they did not re-differentiate into adenocarcinoma in vivo but gave rise to NEPC similar to the castration-resistant relapsed LTL331R tumor. However, distinct from LTL331R, the LTL331_CR_Tumor still expresses AR and does not express additional NE markers beyond CD56. Nevertheless, the NE and AR gene signatures demonstrate that the LTL331_CR_Tumor is NEPC with AR functional loss (Figure 3 and Figure S3). Similar histopathological features have been observed in clinical samples [6,9]. The mechanisms underlying the loss of AR activity in some AR-positive NEPC clinical samples and experimental models remain elusive. Previous studies have shown that SOX2 can induce lineage plasticity and inhibit AR function in PCa cells [52]. In our LTL331_CR models, we found that SOX2 is induced in CR cells and further elevated in CR tumors (Figure S5). This gradually increasing expression is also observed in the LTL331/331R NEPC development model (Figure S5). The increase of SOX2 in CR cells may be unique to LTL331, for it is not induced in CR cells derived from other sources [22]. The precise mechanisms warrant further investigation. The NEPC status of LTL331_CR_Tumor is stably maintained in both castrated mice and in testosterone-supplemented intact mice. Based on our previous PDX in vivo studies, ADT can reproducibly and irreversibly trigger adeno-to-NE transdifferentiation in LTL331 [10,11]. Interestingly, we found that LTL331_CR_Cells mimic the post-castration status of LTL331 at the transcriptomic level. This suggests that the CR culture provides a niche condition for LTL331_CR_Cells, allowing them to gain plasticity and directly differentiate into the NE lineage in vivo. This is not observed in other PCa tumor-derived cells. We speculate that the unique genetic and epigenetic characteristics of the parental LTL331 tumor contribute to this distinct differentiation ability. The underlying mechanisms are being actively studied. Previously, we have used our LTL331/331R model to identify multiple driver genes contributing to NEPC development, including HP1α, PEG10 and ONECUT2 [11,12,15]. With these LTL331_CR_Cells, we can perform on-target genetic manipulation or forward genetic screening to evaluate whether a gene can accelerate or abrogate NEPC development in vivo. We can also perform drug screening on these cells to test whether a drug candidate could block NE differentiation.

## 5. Conclusions

We established CR cells from the LTL331 PDX model. They can be efficiently propagated and genetically manipulated in vitro. LTL331_CR_Cells spontaneously give rise to NEPC tumors in vivo, providing a novel model for studying the mechanisms underlying NEPC development and offering a novel platform for screening drug candidates in a preclinical setting.

## Supplementary Materials

The following are available online at https://www.mdpi.com/2073-4409/9/6/1398/s1, **Figure S1**. Box plots summarizing the different types of single-nucleotide variants (SNV) present in LTL331-dervied samples. **Figure S2**. mRNA expression of *AR* and its target gene *KLK3* as determined by qRT-PCR and RNA-seq. **Figure S3**. (**A**) Neuronal- and proliferation- associated signaling pathways are enriched in LTL331_CR_Tumor compared to LTL331 PDX as analyzed by GSEA. The y-axis represents normalized enrichment scores (NES). The nominal *p*-values of all gene sets are less than 0.05. (**B**) GSEA shows that androgen response is downregulated in LTL331_CR_Tumor compared to the parental LTL331. (**C**) Expression of AR signaling genes in LTL331-derived samples are shown in a heatmap. The samples and genes are clustered by an unsupervised hierarchical method. **Figure S4.** IHC staining of regrafted tumors arising from CR cells derived from a different prostatic adenocarcinoma tissue. Representative images show H&E staining and IHC staining of epithelial tumor marker pan-CK, proliferation marker Ki67, NE marker CD56, AR, and AR signaling marker PSA. Scale bar, 100 μm. **Figure S5**. Expression of SOX2 in LTL331 CR models and PDX tumors as determined by RNA-seq. The y-axis represents log2 transformed relative expression fold-change compared to the parental LTL331 tumor. **Table S1**. Geneset used in Figure 2B. **Table S2**. Cancer hallmarks significantly enriched in LTL331_CR_Cells compared to LTL331_Cas_12 week as analyzed by GSEA.
