# Supplementary material for: Conditionally Reprogrammed Cells from Patient-Derived Xenograft to Model Neuroendocrine Prostate Cancer Development
Source: Cells. 2020 Jun 4;9(6):1398. doi: 10.3390/cells9061398 (PMC7349646; doi:10.3390/cells9061398)
Supplement: Supplementary file 1 [file cells-09-01398-s001.zip › SI.1 Python code.docx]

Coding script used for transcripts mutation calling:

STAR --runThreadN 12 --genomeDir /home/jhao/STAR/hg19 --readFilesIn LTL331-parental_S10_L001_R1_nonmouse1.fastq LTL331-parental_S10_L001_R2_nonmouse1.fastq --outSAMtype BAM SortedByCoordinate --outFileNamePrefix ./LTL331-parental_S10_L001_nonmouse

java -jar /mnt/e/xc/picard.jar AddOrReplaceReadGroups I=./LTL331-parental_S10_L001_nonmouseAligned.sortedByCoord.out.bam O=./LTL331-parental_S10_L001_nonmouseAligned_picard_sorted.bam SO=coordinate RGID= LTL331-parental_S10_L001_nonmouse RGLB=rna RGPL=illumina RGPU=hiseq RGSM= LTL331-parental_S10_L001_nonmouse

java -jar /mnt/e/xc/picard.jar MarkDuplicates I=./LTL331-parental_S10_L001_nonmouseAligned_picard_sorted.bam O=./LTL331-parental_S10_L001_nonmouse_dedup.bam CREATE_INDEX=true VALIDATION_STRINGENCY=SILENT M=./LTL331-parental_S10_L001_nonmouse_dedup.metrics

gatk SplitNCigarReads -R hg19.fasta -I LTL331-parental_S10_L001_nonmouse_dedup.bam -O LTL331-parental_S10_L001_nonmouse_dedup_split.bam

gatk HaplotypeCaller -R hg19.fasta -I LTL331-parental_S10_L001_nonmouse_dedup_split.bam -O LTL331-parental_S10_L001_nonmouse.vcf

perl /mnt/e/xc/annovar/convert2annovar.pl -format vcf4 LTL331-parental_S10_L001_nonmouse.vcf > LTL331-parental_S10_L001_nonmouse.avinput

perl /mnt/e/xc/annovar/table_annovar.pl LTL331-parental_S10_L001_nonmouse.avinput /home/jhao/humandb/ -buildver hg19 -out LTL331-parental_S10_L001_nonmouse -remove -protocol refGene,ALL.sites.2015_08 -operation g,f -nastring . -csvout
