# Supplementary figures and images for: Conditionally Reprogrammed Cells from Patient-Derived Xenograft to Model Neuroendocrine Prostate Cancer Development
Source: Cells. 2020 Jun 4;9(6):1398. doi: 10.3390/cells9061398 (PMC7349646; doi:10.3390/cells9061398)

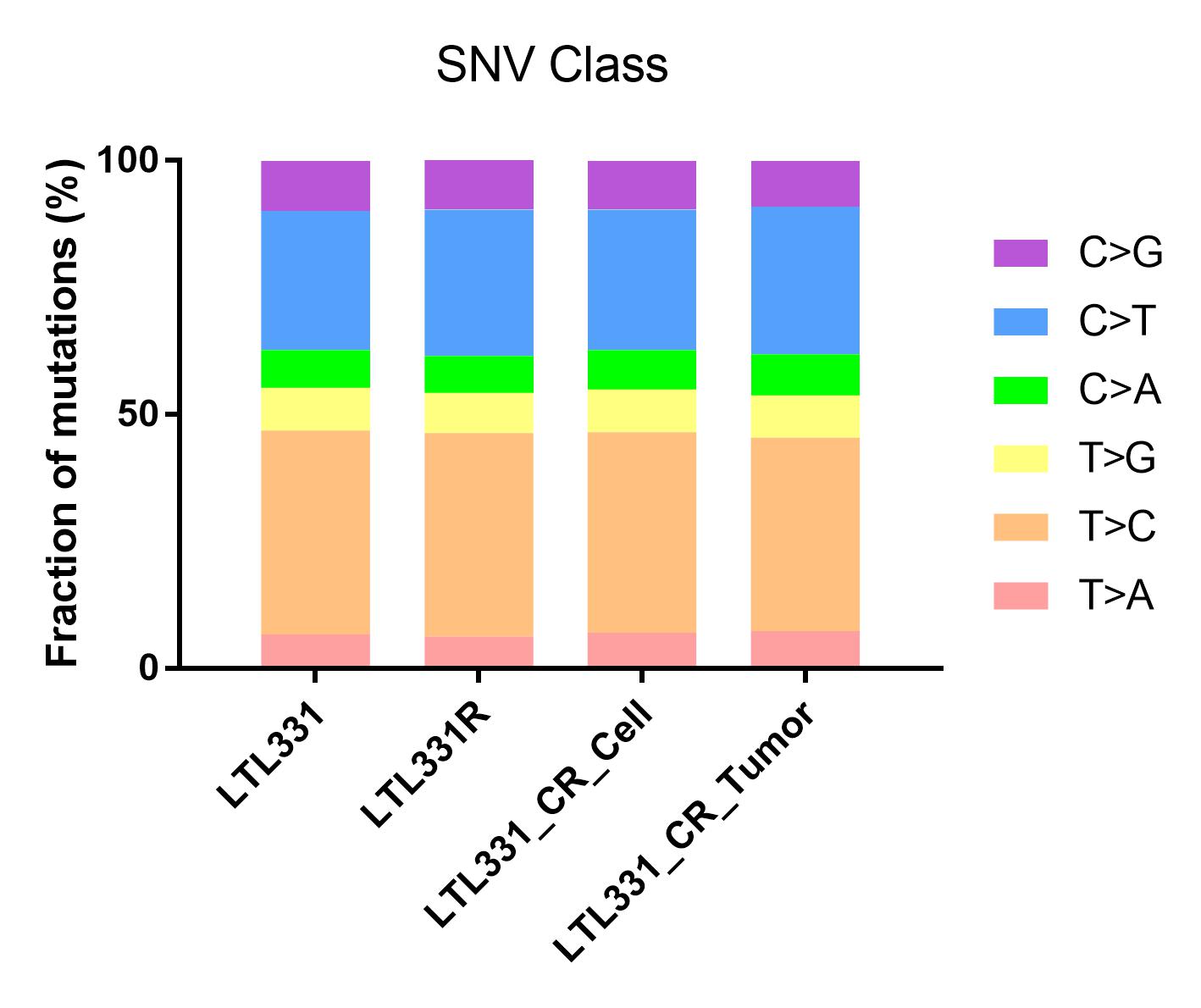

Supplement: Supplementary file 1 [file cells-09-01398-s001.zip › Figure S1.jpg]

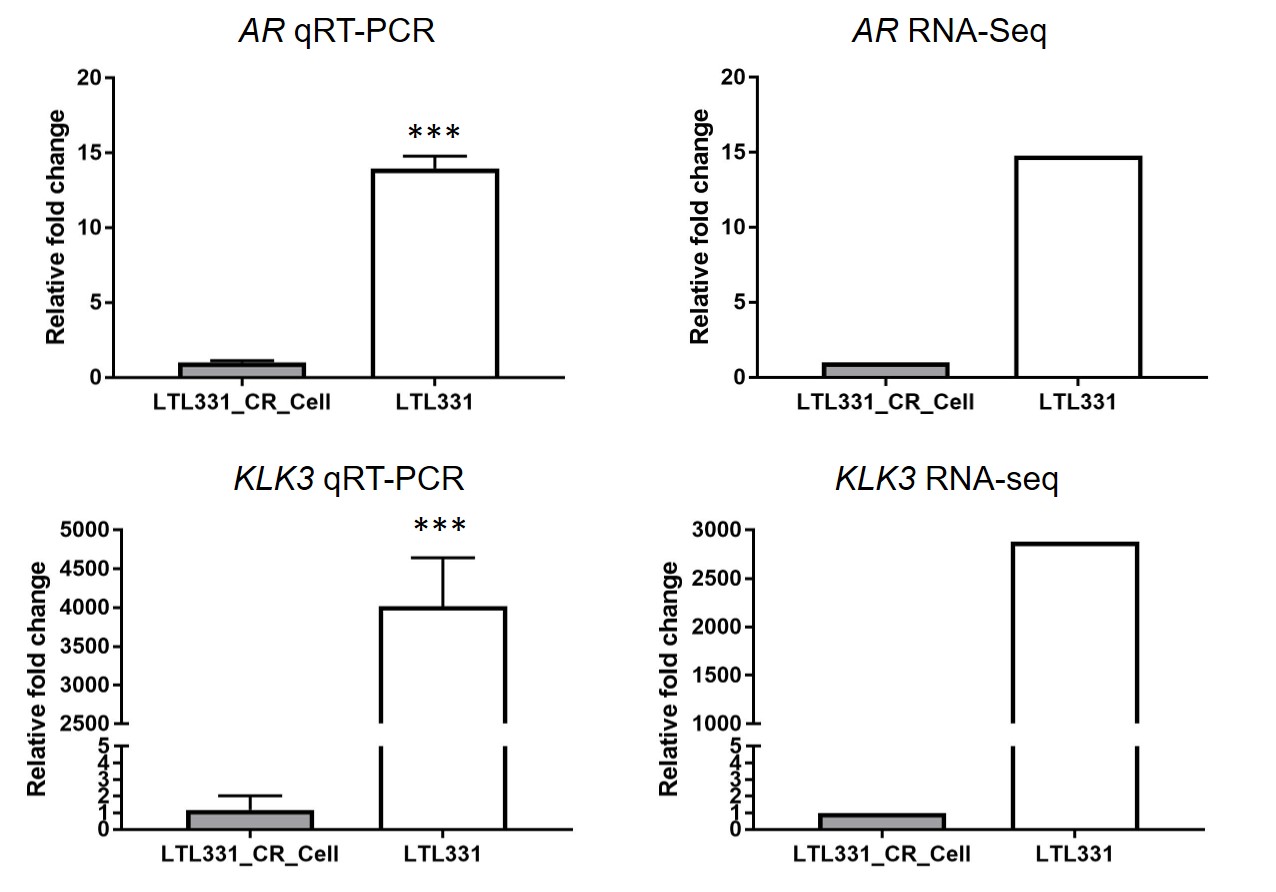

Supplement: Supplementary file 1 [file cells-09-01398-s001.zip › Figure S2.jpg]

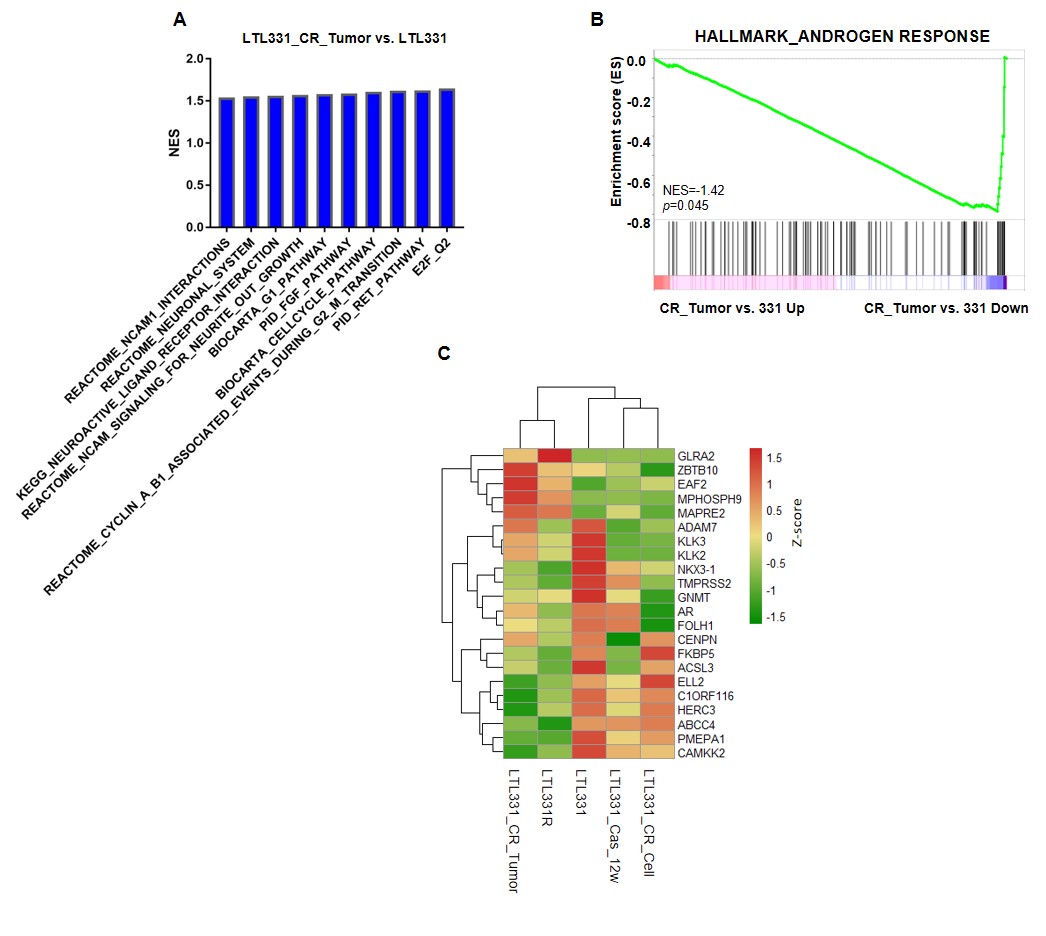

Supplement: Supplementary file 1 [file cells-09-01398-s001.zip › Figure S3.jpg]

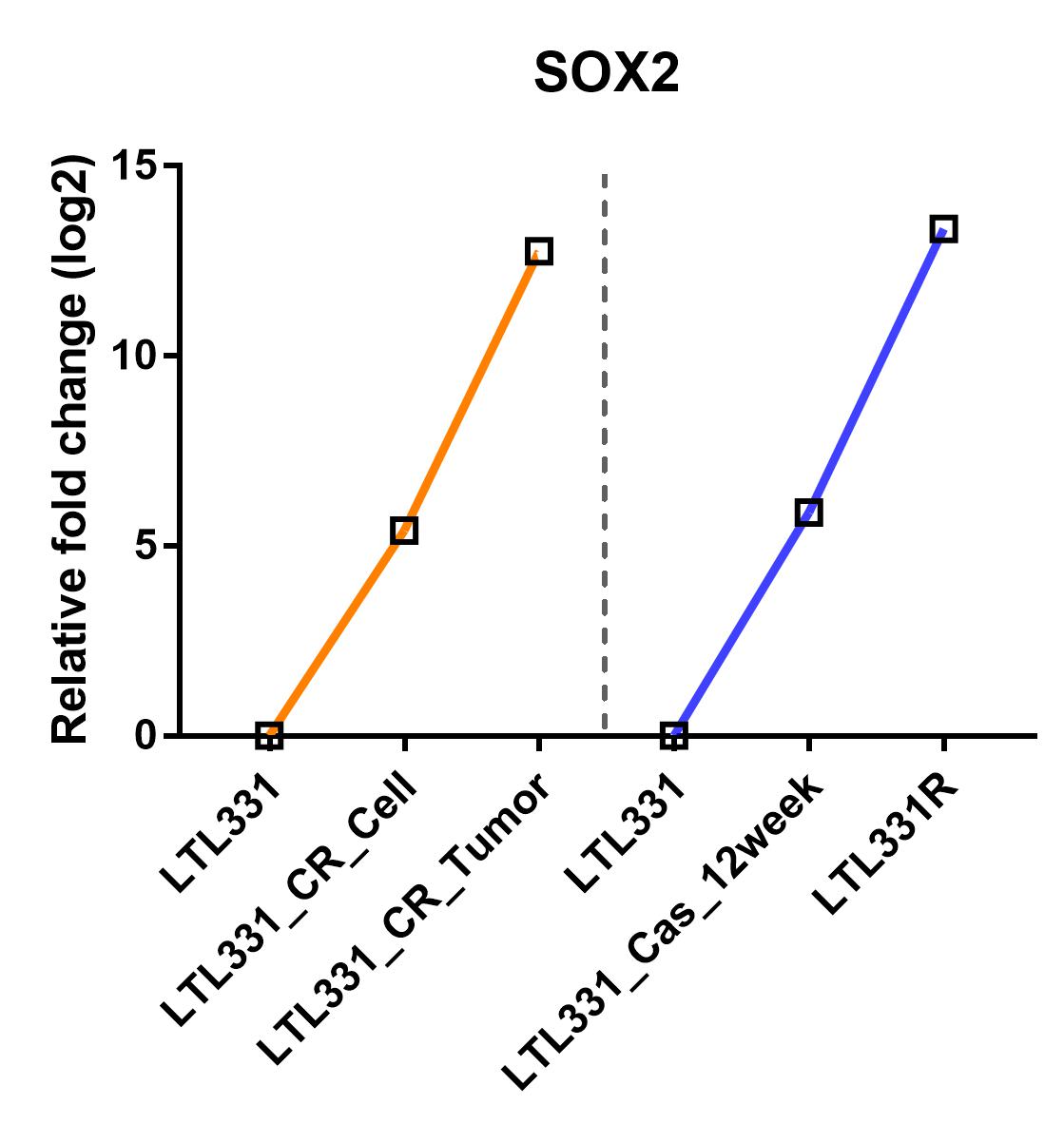

Supplement: Supplementary file 1 [file cells-09-01398-s001.zip › Figure S5.jpg]

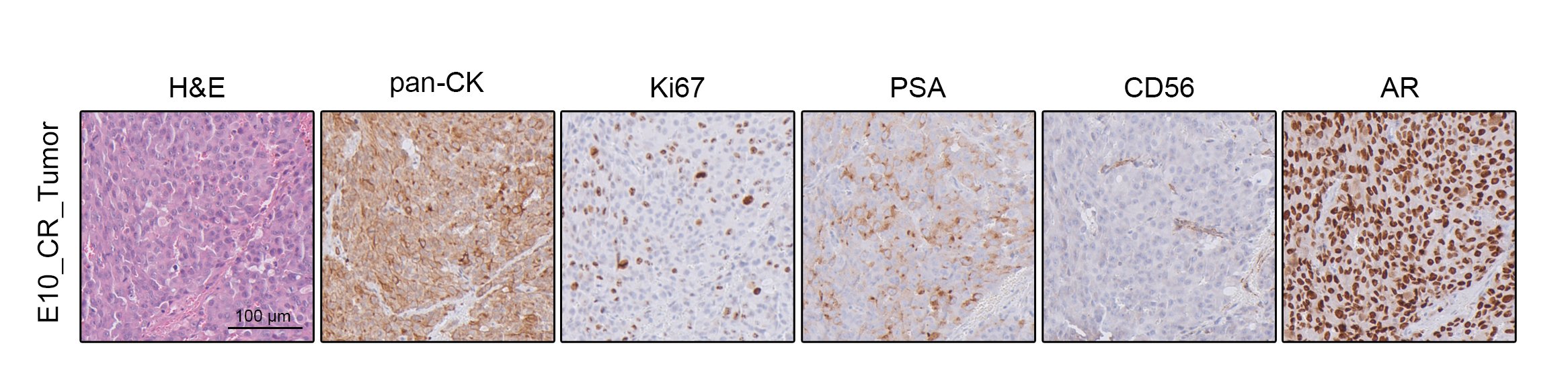

Supplement: Supplementary file 1 [file cells-09-01398-s001.zip › Figures S4.tif]
